# Theoretical Study of Polarization Holographic Encryption via a Nano-Structural Metasurface

**DOI:** 10.3390/nano16060351

**Published:** 2026-03-12

**Authors:** Yingying Tang, Bin Zhang, Zheqiang Zhong, Meihong Rao, Pengyu Zhu, Jiawei Guo, Liancong Gao, He Cai, Dongdong Wang, Hai-Zhi Song, You Wang

**Affiliations:** 1 Quantum Research Center, Southwest Institute of Technical Physics, Chengdu 610041, China; 807255609@qq.com (Y.T.);; 2 College of Electronics and Information Engineering, Sichuan University, Chengdu 610044, Chinazheqiangzhong@scu.edu.cn (Z.Z.); 3 Clinical Medical College, Jilin Medical University, Jilin 132000, China; 4 State Key Laboratory of High Power Semiconductor Lasers, Changchun University of Science and Technology, Changchun 130022, China; 5 Institute of Fundamental and Frontier Sciences, University of Electronic Science and Technology of China, Chengdu 610054, China

**Keywords:** metasurface, information encryption, polarization, nanorods

## Abstract

Metasurface is a kind of artificial structure which can efficiently control the amplitude, phase, frequency, and polarization of the light field. Metasurface polarization holographic encryption is a holographic encryption technology with the polarization state as a key, which has been widely concerned in recent years with advantages such as sub-wavelength pixels, precision adjustment, and high security factor. In this paper, the design and optimization of the unit structure of metasurface have been carried out, and the clear double-channel holographic image reproduction and good encryption effects have been realized afterwards. The results show that the relatively good polarization holographic encryption can be achieved by employing the designed Si nanorods with the length of 148 nm and width of 55 nm, respectively, which have been beforehand grown on SiO_2_ substrates. Note that the periodic angle deflection around the Z axis was adopted by using the dual-channel optical rotation incidence with the wavelength of 632.8 nm. It has been theoretically demonstrated that information transmittance loss should be less and the image restoration effect should be satisfactory. A novel encryption method has also been proposed for the optical information processing and optical encryption, and the huge application potential of our theme has been revealed as the next-generation optical control platform in the near future.

## 1. Introduction

In recent years, the studies in nano photonics have continued to advance, with the optical transmittance and control at the micro- and nano-scales attracting widespread attention. Metasurfaces, as two-dimensional artificial structures composed of the wavelength or sub-wavelength structural units, have recently sparked significant interest due to their compact structure and powerful functionality. They can be effectively used to control parameters such as the polarization, phase, and amplitude of light [1]. In 2011, F. Capasso et al. proposed the use of structurally graded metal antennas to form the periodic structures [2]. By introducing phase gradients, they realized abnormal optical phenomena and deduced the generalized Snell’s law. Since then, studies on metasurfaces have been rapidly developed, with the applications in meta-holography, meta-lens, structural coloration, vortex beams, polarization conversion and polarization beam splitting [3,4,5,6,7,8].

Metasurface is an artificial planar structure that has the superior capability of controlling the depth of field (DOF) of light at the subwavelength scale, leading to a series of novel optical encryption technologies [9,10,11,12,13,14,15]. The key point to metasurface holographic encryption lies in the precise and independent control of multidimensional characteristics of the optical field, such as the phase and polarization state. In 2020, Q. Dai et al. developed a metasurface design strategy that integrates three functions, enabling simultaneous control of spectral characteristics, polarization direction, and phase distribution. However, this strategy still faces challenges in multi-band functionality and the complexity of polarization state control [16]. In 2022, H. Zhou et al. proposed a dual-band vector polarization hologram scheme, in which a visible light hologram and a polarization state key were utilized to unlock encrypted information in the UV band, thereby enhancing security. Nevertheless, the decryption process remains to be complex, and the integration of multi-band functionality is still a challenge [17]. Meanwhile, J. Kim et al. proposed a strategy for constructing dynamic multifunctional metasurfaces using phase-change materials (PCMs) such as Sb_2_S_3_ nanobricks [18]. Such a metasurface, made of Sb_2_S_3_ nanobricks, allows for the simultaneous and tunable control of amplitude and phase. PCMs may exhibit stability issues in practical applications, particularly under high-temperature conditions or over extended periods of use, which could lead to performance degradation. In 2023, S. Q. Zhu et al. introduced a dynamic terahertz metasurface based on a hybrid of vanadium dioxide (VO_2_) and metallic antennas [19]. A thermal control mechanism was employed to achieve optical encryption and decryption in their design process. Although the scheme utilized specific polarization combinations to unlock information, the decryption process remains to be intricate, lacking an efficient and straightforward mechanism.

The previous studies have shown that the complexity of integrating multi-band functions makes it difficult to achieve multiple functions on a single metasurface. At the same time, the limitations of polarization-state control restrict the security and flexibility of an encryption system. Moreover, the decryption process is also complex, lacking an efficient and simple mechanism. Some designs relied on specific materials, which imposed certain limitations on material selection and processing techniques, restricting their applications in large-scale production.

To address these shortcomings, we first proposed an improved Gerchberg–Saxton algorithm (GS algorithm, an algorithm for the rapid solution of the phase of the complete wave function whose intensity in the diffraction and imaging planes of an imaging system are known [20]), which enhances data compactness and transmittance, enabling the encoding and display of multiple images. Then, a procedure of Finite Difference Time Domain (FDTD), a computational technique used to solve partial differential equations, has been used to precisely simulate and optimize the metasurface structure, determining the optimal size and arrangement of nanostructures to achieve the efficient optical encryption and holographic imaging [21]. The simulation tool we used is ANSYS Lumerical FDTD(ANSYS 2023 R2). In this paper, the metasurface multiplexing technology has been introduced to achieve the polarization-keyed multi-channel encryption and display for images, enabling the simultaneous presentation of multiple images on a single metasurface to increase the capacity and information transmittance. The overall design has been based on a polarization-state dynamic information encryption and display structure, which can be used to realize the dynamic encryption and information display by switching circularly polarized light, thereby enhancing security and flexibility [22,23,24,25]. Most of the above studies focus on single-channel information transmission encryption, and the relative processes are somewhat complex. Some of them even require special encryption techniques such as temperature regulation with complicated external structures, which is not conducive to integration. In addition, silicon nanorods on a SiO_2_ substrate were adopted to realize the polarization-sensitive holographic encryption for their excellent thermal and mechanical stability. What is more, silicon nanorods are highly compatible with existing semiconductor manufacturing processes, significantly enhancing the manufacturability and reliability of metasurface structures. Through theoretical evaluation, the application performance of metasurfaces can be significantly enhanced in the fields of optical encryption and multi-channel information display by using our approaches such as improved algorithms, optimized structural design, and the introduction of polarization-state dynamic encryption, providing a new route for the development and application of metasurface technology. The fine control ability of metasurfaces has been conducted over the degrees of freedom of light waves, precisely encoding incident light waves at different polarization angles to construct multiple images with clear layers. It has been demonstrated that metasurfaces can be used for the construction of the next-generation light control platform in the development of optical information processing and optical encryption. Until now, similar studies have rarely been published in the field of encryption and information display, to the best of our knowledge.

## 2. Design Principles and Simulation

The left- and right-circular polarization algorithms have been adopted to improve the original GS algorithm. Compared with the original basic GS iterative algorithm, the superiority of the new algorithm lies in the use of dual channels, which improves data compactness and the efficiency of single data transmittance. The topic of this paper is phase recovery and image reconstruction.

### 2.1. Algorithm Design and Implementation

Phase recovery is mainly achieved through the GS algorithm. Generally, the GS algorithm mainly focuses on the phase and image planes. First, a random phase was utilized to assign the phase information of two inputted images, SCU and META, to accelerate the convergence. Subsequently, two new complex amplitudes were formed using the assigned phase information, and the imaging plane was constructed via Fresnel diffraction. On the imaging plane, only the phase information was retained while the associated amplitude information was discarded, and the amplitude was set to 1 to form a new complex amplitude. Thereafter, a process of inverse Fourier transform (IFT) was performed to return to the phase plane. When the number of iterations reached the set threshold, the program converges and exits the iterative loop, ultimately obtaining the desired phase information.

**Figure 1:**
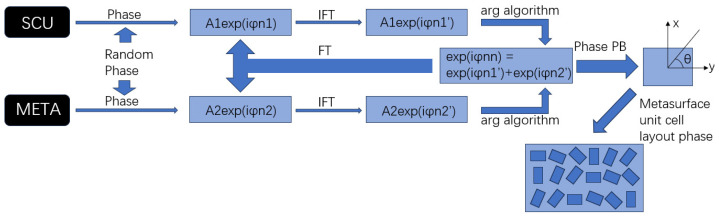
Illustration of computation for the unit cell layout on metasurface.

The overall concept of the algorithm is shown in Figure 1. The lower phase information was extracted from two-channel inputted data through the GS algorithm, which is called “arg algorithm”, to be used for the calculation of the complete phase information for subsequent nanorod encoding design of the metasurface. The total phase information was analyzed by using the PB phase principle to determine the corresponding tilt angle of the unit nanorod. Finally, the periodic arrangement has been performed so that the metasurface structure can carry the initial information, waiting for appropriate incident light to decode the originally encoded image information in the far-field hologram.

### 2.2. Structural Design and Implementation

**Figure 2:**
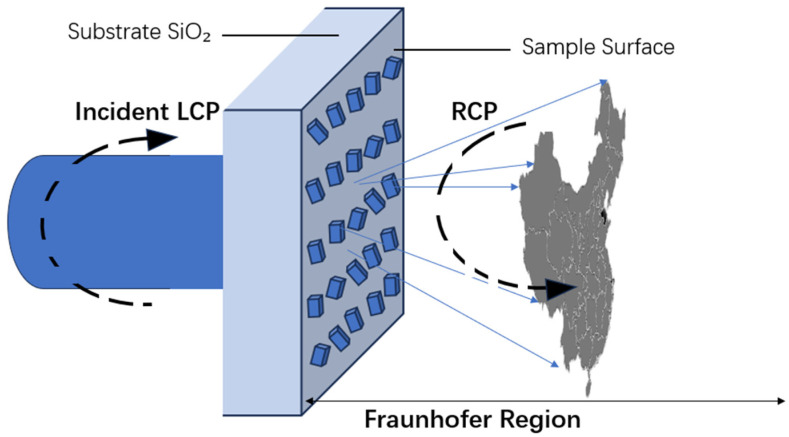
Schematic illustration of holographic images.

**Figure 3:**
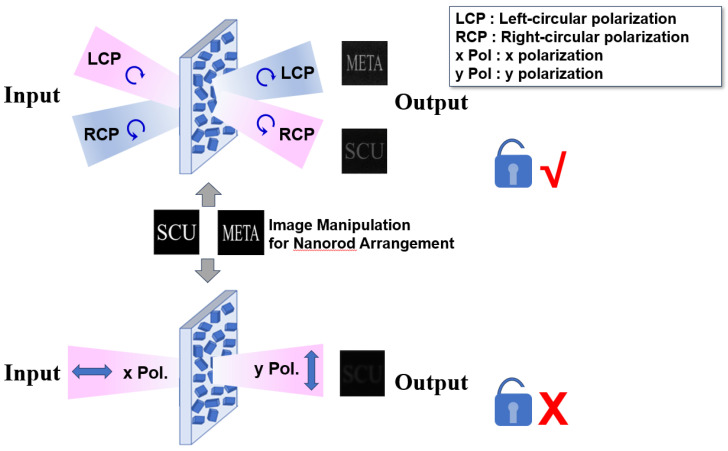
Illustration of a decryption process with two channels.

In the schematic diagram as shown in Figure 2, the process of left-circularly polarized (LCP) light is depicted for the illustration of a light wave entering from the left side onto the sample surface. Such a process aims to achieve precise control of light waves through some carefully designed metasurface samples. Subsequently, the controlled light exits as right-circularly polarized (RCP) electromagnetic waves. The desired holographic image can be formed within a Fraunhofer diffraction region of holographic samples [26]. Also, the control mechanism relies on a series of carefully arranged elongated nanorods with different rotation directions to achieve fine control of the polarization state of light waves. Figure 3 reflects the illustration of a decryption process with two channels. The original image information has been used to modulate the arrangement of the nano-columns of the metasurface. When left circularly polarized light is incident and right circularly polarized light is emitted, the image “SCU” of channel one can be decoded. When right circularly polarized light is incident and left circularly polarized light is emitted, the image “META” of channel two can be decoded, proving that the decryption process is successful. However, when the incident polarization state does not match the required decoding key, the output image cannot be decoded. Taking the X-direction polarized light as an example, the emitted light exhibits Y-direction polarized state. At this time, the emitted light is very weak and the image is almost invisible, indicating that the decryption has failed.

Through the above design, we not only demonstrated the powerful capability of metasurfaces during controlling light wave but we have also verified the effectiveness of the GS algorithm in holographic images with the complex light field, providing new ideas and methods for the studies in optical encryption, information storage, and display technology.

**Figure 4:**
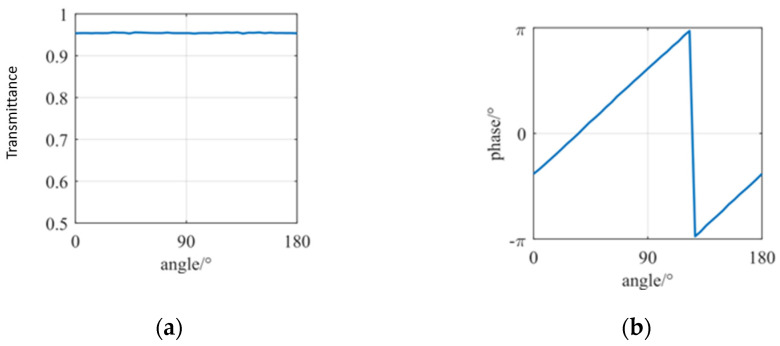
Simulation results of the transmittance and phase vs. the nanorod rotation angle θ: (**a**) transmittance; (**b**) phase.

During the calculation of Figure 4, the sizes of the length and width are both selected to be 50 nm. Throughout the scanning of the nanorod angle θ from 0° to 180° in the Z direction, the transmittance rate changes a little, with the overall curve trend being smooth. The transmittance rate can be basically stabilized at around 0.954. It reaches a peak at the angle of 50° and a trough at around 130°, with the lowest value not lower than 0.953 and the highest value not higher than 0.956, which proves the robustness of the angle. When the orientation angle of the nanorod changes, the transmittance rate will fluctuate. But, since the hologram has tolerance to amplitude noise, the fluctuation can be ignored. As can be seen from Figure 4b, during the scanning process, the phase reaches −1.2 at 0° and 180°, and reaches a peak and trough at around 130°. The overall trend of the image is relatively steep. In general, the phase can cover the range of 2π, providing phase support for the hologram. Therefore, the requirements for the phase distribution of the hologram and the usage conditions for the PB phase principle can be both satisfied.

**Figure 5:**
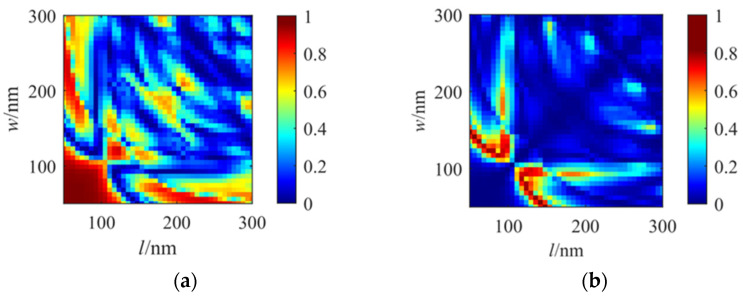
Simulation results of transmittance distribution corresponding to different lengths and widths of a nanorod: (**a**) LCP transmittance; (**b**) RCP transmittance.

**Figure 6:**
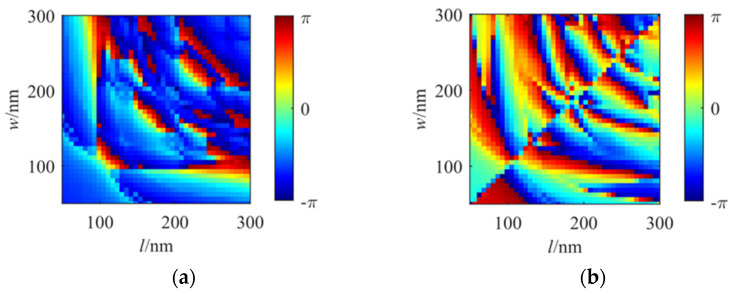
Simulation results of phase corresponding to changes to different lengths and widths of a nanorod: (**a**) LCP phase; (**b**) RCP phase.

The results of the dimensional group scanning are shown in Figure 5 and Figure 6. When the length and width of nanorods are simultaneously changed, due to the properties of the incident light in the left and right channels, the images exhibit a symmetric distribution. Some analyses have been performed along the diagonal axis of symmetry. Moreover, two images in Figure 5a,b exhibit complementary effects. Points in Figure 5a reaching the maximum values correspond to minimum values in Figure 5b. In Figure 5b, the transmittance rate can reach 0.818, shown as the bright red area in the figure, corresponding to dimensions with the length of 0.148 μm and width of 0.055 μm, respectively. In summary, the final dimensions of the unit nanorod have been selected as L = 0.148 μm and W = 0.055 μm in the simulation.

In this paper, the theories and simulation are based on the following two assumptions: (1) when the left circularly polarized light is incident, the polarization state of light after passing through the metasurface is partly converted to the right-circular polarization; (2) similarly, when the right circularly polarized light is incident, the polarization state of light after passing through the metasurface is partly converted to left circular polarization. Actually, after part of the left circular light is converted to right circular light, the remained part still presents the left circular polarization state.

**Figure 7:**
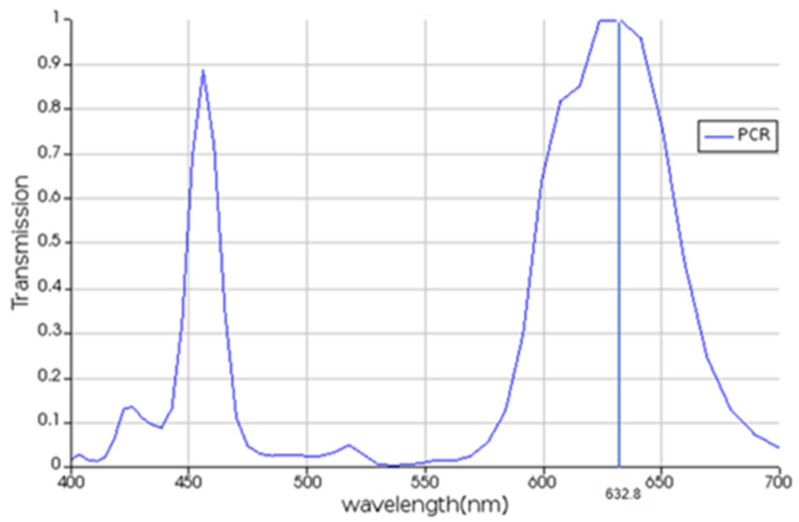
PCR analysis results.

Therefore, the wavelength with the highest polarization conversion rate (PCR) needs to be selected for the simulation. The calculation formula is

(1) $$PCR\;=\;{LR}^{2}\;/\;({LR}^{2}\;+{LL}^{2}),$$

where LR means the rate of the left circular light that has converted to the right circular light, and LL means the rate of the remained part of the left circular status, respectively. Whether the 632.8 nm wavelength used in the simulation can achieve the maximum conversion rate or not has been verified in this paper. As shown in Figure 7, PCR reaches peaks at two points: one is located at 470.0 nm and the other is located at 632.8 nm, confirming the correctness of the simulation wavelength selection.

**Figure 8:**
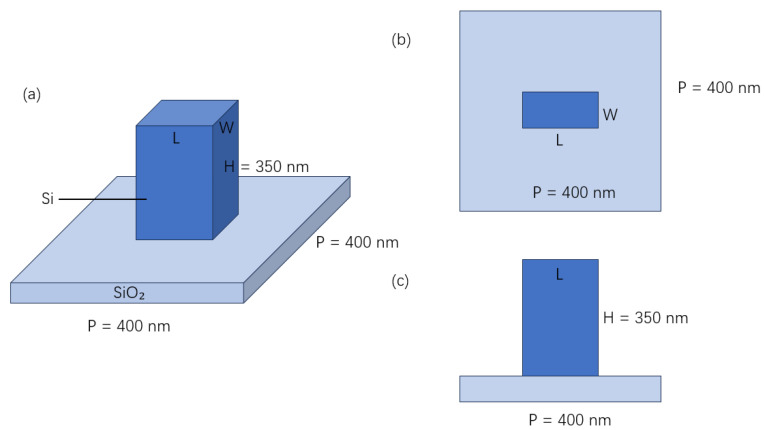
Illustration of a unit in the theoretical design: (**a**) overall structure; (**b**) top view; (**c**) side view.

In this paper, the unit structure is designed as a configuration in which Si nanorods are formed on a SiO_2_ substrate, as illustrated in Figure 8. To achieve the optimal transmittance, a procedure of FDTD has been used in the simulation of nested scanning of both the length L and the width W. Images have been applied to intuitively understand the maximum transmittance which can be achieved by employing the current structure, and data matrices have been exported and processed to calculate the maximum point, which serves as an optimal dimensional solution. When selecting the material for the construction of nanorods, considering that metal materials would result in significant ohmic losses, Si was chosen to avoid ohmic losses, thereby improving the optical performance. The substrate material was selected as SiO_2_, with the single period length of *p* = 400 nm to avoid the higher-order diffraction, and the height H of 350 nm to ensure the corresponding phase delay. During the simulation of the unit structure, the frequency domain solver is commonly used as a computation tool. Generally, the boundary conditions in a “unit cell” have been applied to simulate periodic structures in horizontal x and y directions. In this paper, the designed metasurface structure has been set to be periodic during the FDTD simulation. The simulation wavelength was chosen as 632.8 nm, which was thought to be suitable for the simulation of simulation. The incident circularly polarized light can be combined using two mutually perpendicular linearly polarized beams with the phase difference in ninety degrees. As shown in Figure 3, the left circularly polarized light and the right circularly polarized light were assumed to be incident in Channel 1 and Channel 2, respectively. The target images cannot be obtained if the polarization status is exchanged or other polarization angles are used. Finally, the dimensions of the unit nanorod have been determined as L = 0.148 μm and W = 0.055 μm, respectively.

## 3. Result Analyses and Discussions

### 3.1. Test Results with GS Algorithm

**Figure 9:**
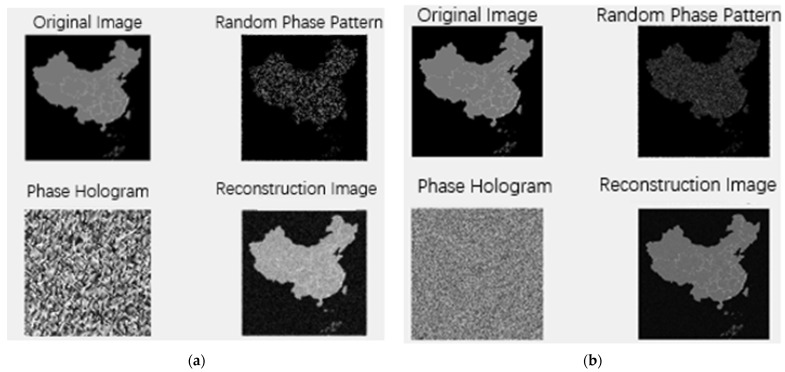
Original and holographic reconstructed images: (**a**) 100 × 100 resolution; (**b**) 500 × 500 resolution.

The theoretical holograms during the simulation of the GS algorithm are shown in Figure 9. At the resolution of 100 × 100, the effect is slightly inferior, with blurred details. However, the overall contour information is still clear and sufficient for the purpose of information transmittance. It is mainly manifested as clear and distinguishable provincial divisions. When the resolution is set to 500 × 500, a good reconstruction effect can be observed. The top-left image is the original, the top-right is the random phase map, the bottom-left is the phase hologram, and the bottom-right is the reconstructed image, respectively. At a high resolution of 500 × 500, the reconstructed image can present most of the important details of the original image. Note that the performance degradation might be caused by insufficient spatial sampling due to the low resolution and inability of periodic boundary conditions (PBCs) to reflect edge effects in finite-sized metasurfaces.

**Figure 10:**
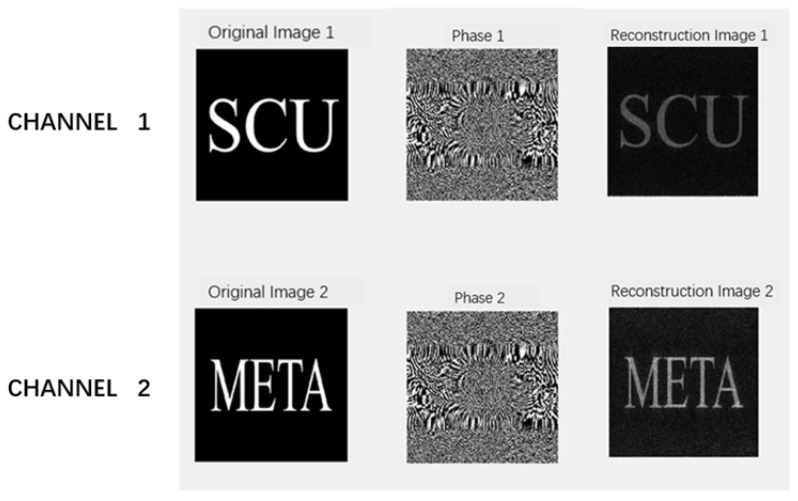
Original images, holographic phases, and reconstructed images for Channel 1 and Channel 2 at 500 × 500 resolution.

Each nanorod with optimized dimensions and orientation acts as a diffractive element pixel, producing the desired local amplitude and phase profile under normal incident light. Two independent images can be displayed under different combinations of light polarization, utilizing the polarization state of the incident light to achieve image encryption display. In our study, Channel 1 was used to display the image from the left circularly polarized light, achieving the effect of decryption. However, if the right circularly polarized light or light with other polarization angles is inputted in Channel 1, the information cannot be decoded, thereby realizing the process of encryption. Similarly, in Channel 2, the right circularly polarized light was used as the incident signal to achieve the effect of decoding the information. If the left circularly polarized light or light with other polarization angles is inputted, the information cannot also be decrypted. Figure 10 shows the process from original inputted images to reconstructed ones using a Double-GS Dual-channel algorithm at the resolution of 500 × 500. Original images 1 and 2 are the images inputted into Channel 1 and Channel 2, respectively. Note that Phase 1 and Phase 2 are respectively the corresponding phase holograms for Channel 1 and Channel 2, and rebuilt Images 1 and 2 are the reconstructed far-field holograms.

### 3.2. Test Results with FDTD Algorithm

**Figure 11:**
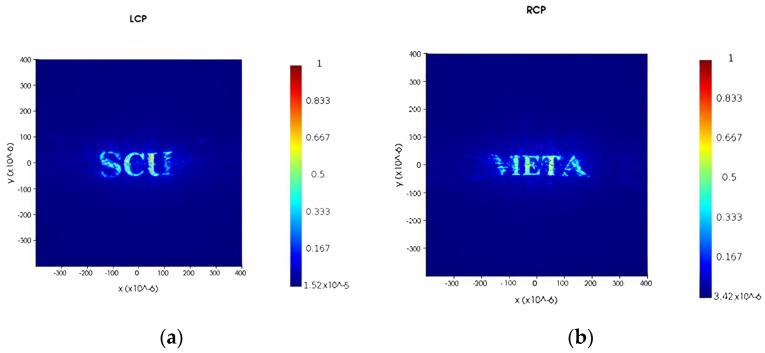
Holographic multiplexing images obtained by the FDTD simulation: (**a**) holographic image outputted from Channel 1; (**b**) holographic image outputted from Channel 2.

From the rebuilt results in Section 3.1, it has been observed that at the resolution of 500 × 500, good holographic reconstruction can be realized by using the GS protocol, clearly restoring most of the details of original images with almost no significant information losses. Theoretically, increasing the resolution under the current metasurface structure can improve the quality of reconstructed images. In fact, there are differences between GS and FDTD algorithms. The edge clarity of the GS results is better than that of the FDTD ones, but the contrast of images reconstructed by the FDTD procedure is stronger than that reconstructed by the GS one. Scripts were used in FDTD to periodically arrange the unit nanorod structure with sequentially rotated angles. Under infinite periodic boundary conditions, script parameters and materials have been set, and the far-field hologram can be therefore obtained. The realization effect has been demonstrated for the far-field holograms under periodic simulation arrangement. The far-field holographic reconstruction results from the FDTD simulation are shown in Figure 11 for periodically arranged unit structures. Limited by computational resources, the resolution of 100 × 100 is used in the simulation. The letters “SCU” in Channel 1 can be clearly recognized, although the top and bottom of the initial letter “S” are slightly unclear, and the bottom-right edge of the trailing letter is blurred. In Channel 2, the left half of the initial letter “M” is somewhat missing. Overall, the letter information can still be correctly identified. However, the actual performance of the metasurface structure cannot fully restore the theoretically forecasted clarity by the use of both the GS and the FDTD protocols.

### 3.3. Discussions

From the simulation results in Section 3.1 and Section 3.2, the GS and FDTD algorithms can both be used to complete information reconstruction and transmit the detailed information of the original image. The overall conceptual framework of this design process is configured in a dual-channel operation mode. In fact, the polarization encryption exhibits the unique advantages of polarization multiplexing technology in achieving dual-channel holographic display. Such mechanism allows the system to process the mutually independent information from two channels in parallel, i.e., each channel is only sensitive to the light of a specific polarization state, effectively ensuring the independence and security of the information. When the unauthorized light sources are used, any meaningful information cannot be extracted even if the receivers have advanced phase recovery ability, which greatly improves the efficiency and confidentiality of data transmittance.

By cleverly determining parameters such as the amplitude, geometric morphology, and propagation phase to design a single metasurface structure, the information encoding density and display flexibility can be achieved in this scheme. Simulation results show that during the design process of this metasurface, not only two independent information channels have been successfully encoded and stored, but also high information capacity and excellent security performance have been realized, ensuring the integrity and immutability of the information. One can see that the decoding process requires precise matching of specific polarization light combinations, further consolidating the security barrier of our system.

## 4. Conclusions

An innovative polarization holographic encryption strategy has been proposed in this paper. The research core is to use the designed Si nanorod array as the structural basis to achieve a highly integrated information encryption and decryption. This study focuses on the design of periodic nanorod metasurfaces, achieving a polarization-sensitive holographic encryption system and demonstrating the reconstruction effects for information-rich holographic images even under resolution-limited conditions. Simulation results have shown that our design exhibits good performance under the existing computational conditions. One can expect that if computational support becomes better, higher resolution and better quality of holographic images could be achieved. Taking metasurface lenses for instance, high resolution has been achieved during imaging with the neural-network-assisted metalens, which is comparable to that of the ground truth image [27]. Furthermore, through comprehensive evaluations on diverse synthetic and real-world data sets captured under various environmental conditions and focusing distances, the significant enhancements in image quality have been consistently demonstrated through the approach developed by Y. Dong et al. [28]. In this study, the relatively good polarization holographic encryption effects have been proved by the use of the metasurface in which some designed Si nanopillars with the length of 148 nm and width of 50 nm, respectively, are arranged on SiO_2_ substrates. Note that the periodic angle deflection around the Z axis has been adopted by using the dual-channel optical rotation incidence with the wavelength of 632.8 nm. It has been demonstrated that information transmittance loss is less and the image restoration effect is in accordance with the theoretical expectations. The broad applications of this research might be found in the field of information security due to convenience, efficiency, and ease of implementation of our methodology. In terms of the security level, due to the specificity of the polarization decryption channel, the dual-channel polarization encryption scheme has certain security. However, even with incorrect polarization state incidence, the outputted image should still contain some components of the dual-channel original images. Further studies are being undertaken to strengthen confidentiality to achieve the effect without any image leaking.

Nevertheless, current technology faces some challenges in material performance. For example, although the selected material has achieved the transmittance rate of 0.813, exploring new optical design strategies and finding high-performance materials should be useful to fundamentally address the limitations of existing technologies. Despite the fact that the optimization of the unit structure of metasurface has been carried out, promising potential could be harnessed for further optimization of decryption approaches. Additionally, to enhance the capacity and flexibility of single information transmittance and break through the limitations of the existing key system, one can also increase the number of independent channels with more polarization statuses of inputted light in the near future.

## Data Availability

The original contributions presented in this study are included in the article.
